# Circular saw blade wear status prediction based on generative adversarial network and CNN-LSTM model

**DOI:** 10.1371/journal.pone.0326044

**Published:** 2025-06-18

**Authors:** Chao Zeng, Chengchao Wang, Xueqin Xiong, Xiangjiang Wang, Sheng Xiao

**Affiliations:** 1 School of Nuclear Science and Technology, University of South China, Hengyang, China; 2 Hunan Metallurgical Planning and Design Institute Co., Ltd., Changsha, China; 3 Shandong Iron and Steel Co., Ltd., Jinan, China; 4 School of Mechanical Engineering, University of South China, Hengyang, China; Gazi Üniversitesi: Gazi Universitesi, TÜRKIYE

## Abstract

Monitoring the status of circular saw blades is an effective measure to ensure the production efficiency and safety of spent fuel assembly cutting. However, the prediction of wear during the cutting of stainless steel shells of spent fuel assemblies by circular saw blades is not entirely accurate in complicated working conditions. The main challenges include deficiency of data acquisition, signal features extracting complex process, and insufficient robustness of models. To enhance the precision of forecasting consequence, a circular saw blade wear prediction method combining generative adversarial network (GAN) and CNN-LSTM models is proposed. The main fault types during the cutting of stainless steel shells by circular saw blades should be identified in advance, and vibration signals of each fault state is able to be collected then. The collected data is supposed to be preprocessed through sampling overlapping, single-layer wavelet transform denoising, and normalization. GAN optimized by the Pearson correlation coefficient (PCC) has been utilized aiming to expand the data volume of each fault state to 300 samples and resulting in a total data volume of 2100 samples; A CNN-LSTM model based on dual feature fusion has been established to identify the wear status of circular saw blades, achieving an accuracy rate of 100%, higher than Long Short-Term Memory (LSTM) neural networks (86.2%) and Radial Basis Function Neural Networks (RBFNNs) (94.9%). This study effectively solves the problem of small sample sizes for circular saw blade wear data, and provides an efficient and accurate method for circular saw blade wear identification under complex working conditions, which has important practical significance for improving the safety and efficiency of spent fuel assembly cutting.

## 1 Introduction

In regard to nuclear industry, principles of the selection about cutting tools such as automation, minimization of waste, and strong resistance to radiation must be considered [1,2]. Normally, circular saw cutting is deemed to be as a leading method because its design-friendliness to numerically controlled fully-automatic systems and the low-level generation of radioactive aerosols. Researchers are currently designing the application of circular saw blades to cut stainless steel shells of fast reactor spent fuel assemblies. But during the cutting process, mechanical failure of circular saw blades, such as wear and fracture, happens occasionally. If these issues can’t be promptly identified and addressed, the entire reprocessing workflow may be impacted and some severe incidents might occur at worst [3]. Therefore, the establishment of an intelligent monitoring system for the wear of circular saw blade can significantly improve operation safety and production efficiency, making the study become a research emphasis in related fields.

The primary methods for monitoring tool wear status are divided into direct and indirect methods [4]. The direct method involves directly detecting changes in the geometric shape of the tool through optical measurement or machine vision to assess tool wear. However, direct methods require expensive optical instruments and are difficult to implement in harsh processing environments, especially under radiation conditions [5,6]. On the other hand, indirect methods monitor signals related to tool wear, such as cutting forces, vibrations, temperatures, spindle power, and sounds [7–9]. Machine learning techniques are then employed to extract and classify signal features, enabling intelligent monitoring of tool wear. This approach has been widely adopted [10–12]. For example, Zhou et al. developed a two-layer kernel extreme learning machine (TAKELM) for tool condition prediction based on extracted acoustic sensor signal features [13]. Paweł Twardowski et al. collected acoustic emission signals during the workpiece processing and input them into a decision tree to identify milling cutter wear levels [14]. Studies have also collected cutting force and vibration signals during the processing and utilized optimized support vector machines for tool wear monitoring [15–17]. Wu et al. used a random forest algorithm (RF) to predict milling cutter wear and found that this model outperformed single-hidden-layer neural network models and support vector regression (SVR) models [18]. These methods combine artificial intelligence shallow learning models with sensing technology to provide solutions for tool wear prediction. However, the feature extraction method of shallow learning models is limited by human experience, resulting in low modeling efficiency and time-consuming efforts. In recent years, deep learning, which has deeper network layers and can automatically extract features, has become a research hotspot [19–21]. Zhang et al. established a tool wear monitoring method based on Convolutional Neural Networks (CNN), achieving accurate prediction throughout the entire tool life cycle [22]. Wang et al. proposed a multi-task learning approach utilizing Deep Belief Networks (DBN), and experimental results demonstrated that this model achieved a prediction accuracy of 99% for tool wear and 92.86% for part surface quality [23]. Lai et al. introduced an improved end mill wear identification method based on an interpretable physics-guided spatial attention mechanism, effectively enhancing wear identification accuracy across various process parameters [24]. Ashish Manwar et al. developed a tool condition prediction model leveraging Long Short-Term Memory (LSTM) technology, extracting cutting force features for tool wear monitoring [25]. Additionally, the time series modeling domain has witnessed the emergence of several new Machine Learning (ML) models, such as LSTM-ALO [26], LSTM-INFO [27], and RVM-IMRFO [28]. However, single deep learning networks may face issues such as inadequate feature extraction and poor model robustness [29,30]. Therefore, multi-model combinations has been expected to be applicated extensively. Zhao et al. proposed the use of dual-path parallel CNNs for feature extraction and fusion of sound signals and workpiece surface images, resulting in higher tool wear recognition accuracy compared to single-signal recognition [31]. Huang et al. introduced a tool wear prediction method based on multi-information fusion and Genetic Algorithm (GA) optimization, with experimental results showing that the model exhibited stronger resistance to environmental noise interference [32]. Wang et al. proposed the Multi-Scale Convolutional Attention Network (MSCAN) model, inputting multi-sensor monitoring data from milling tool life tests into the MSCAN model, compared to other prediction models, this model provided more stable tool wear predictions [33]. Li et al. presented a tool wear prediction model based on Temporal Convolutional Long Short-Term Memory (TCN-LSTM) with multi-domain feature fusion, achieving an average prediction goodness-of-fit of 0.96 and superior accuracy and generalization capabilities compared to other models [34]. Zheng et al. established a high-precision recognition model by transforming the feature space of the original signals through Empirical Mode Decomposition (EMD), Variational Mode Decomposition (VMD), and Fourier Transform [35]. Pan et al. fused CNN with Bidirectional Gated Recurrent Unit (BiGRU) to extract spatial and temporal features from vibration signal data, enabling the extraction of richer high-dimensional features and enhancing the algorithm’s feature mapping capabilities [36]. In recent years, many scholars have combined CNN and LSTM networks for tool wear prediction, leveraging CNN’s strong feature extraction ability and LSTM’s memory capability [37]. LYU et al. proposed a semi-supervised parallel gated CNN-LSTM micro-milling tool wear state monitoring method based on wavelet denoising, achieving a tool wear classification accuracy of 93.61% [38]. JIANG et al. extracted multi-domain features from axial force and acoustic emission signals during drilling of CFRP/TC4 laminated structures, inputting them into the CNN-LSTM model to achieve a recognition accuracy of 97.22% [39]. However, the current monitoring models mainly focus on single-edged machining processes such as milling and drilling, making the modeling of multi-edged tools as circular saw blades not able to cover the comprehensive conditions entirely.

In practical applications, collecting large amounts of data for training deep learning models poses a significant challenge. Generative Adversarial Networks (GANs), due to their powerful data generation capabilities, have been widely utilized to address issues related to small sample sizes [40,41]. For instance, Zhu et al. combined finite element simulation technology with GAN models to expand the collection of cutting force signals from cutting tools [42]. Shah et al. constructed a Singular GAN to extend the scale diagrams of acoustic emission and vibration signals related to tool wear signals [43]. Molitor et al. employed various types of GANs to enhance data, resulting in an approximate 18% improvement in the accuracy of tool wear monitoring [44]. Although these GAN-based data augmentation methods have expanded the experimental data on tool wear, they lack evaluation criteria for assessing the training performance of GAN models. Rana et al. proposed Comprehensive Accuracy (CA) as an evaluation metric for assessing model performance [45,46]. Wang et al. attempted to address the limitations of conventional generative adversarial networks (GANs) by incorporating the K-nearest neighbor (K-NN) algorithm, which achieved notable improvements in performance. However, the integration of the K-NN algorithm increased model complexity and resulted in prolonged training processes [47]. Li et al. developed a Bayesian optimization gradient penalty-based Wasserstein GAN for data augmentation, demonstrating a 1.11% enhancement in classification accuracy compared to non-augmented baselines [48]. Zhang et al. introduced a novel regularization technique to stabilize GAN training, significantly improving the model’s classification accuracy from 86.758% to 92.611% [49].

Circular saw blades serving as crucial tools for cutting the stainless steel shells of spent fuel assemblies, features a large number of teeth in its blades which poses challenges due to the scarcity and instability of fault data collected. Furthermore, in irradiated environments, signal acquisition is difficult, not to mention the significant interference, which makes this process not stable enough when using traditional deep learning models for tool wear prediction. Therefore, this paper proposes a wear prediction method for circular saw blades based on Generative Adversarial Networks (GANs) and Convolutional Neural Network-Long Short-Term Memory (CNN-LSTM) models. This method utilizes Pearson correlation calculations to optimize GANs to enhance their data generation performance and strengthen the correlation between generated data and actual data, so the issue of insufficient fault data for circular saw blade wear can be addressed effectively at last. Additionally, a CNN-LSTM model based on dual-feature fusion is constructed to extract the spatiotemporal features of signals efficiently, solving the problems of gradient vanishing and difficult feature extraction. The remaining structure of this paper is as follows: Section 2 presents the theoretical background; Section 3 outlines the framework for circular saw blade wear prediction; Section 4 discusses the experiments and model validation; and Section 5 summarizes the paper.

## 2 Theoretical background

### 2.1 Generative Adversarial Network (GAN)

**Fig 1:**
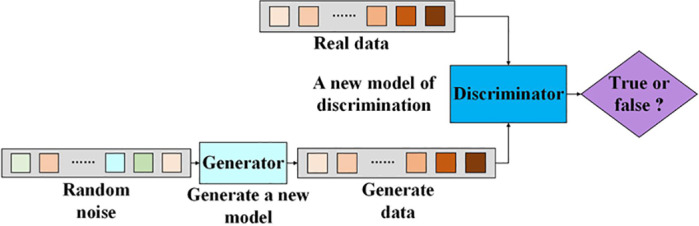
Generative Adversarial Network.

Generative Adversarial Network (GAN) is a deep learning model comprising a generator and a discriminator. This model generates data through the generator and verifies the generated data using the discriminator to produce data similar to real data, thereby achieving the purpose of augmenting experimental data [50]. The training process is illustrated in Fig 1.

The model definition of generative adversarial networks is shown in equation (1).


$$\underset{G}{\min}\underset{D}{\max}V(D,G)=E_{x\tilde{P_{data}}(x)}\left[\log D(x)\right]+E_{z\tilde{P_{z}}(z)}\left[\log(1-D(G(z)))\right]$$ (1)

where $\begin{array}{c} \underset{G}{\min}V(D,G) \end{array}$ is generator loss, $\begin{array}{c} \underset{D}{\max}V(D,G) \end{array}$ is discriminator loss, *G* is the generator, *D* is the discriminator, *x* is the real image, *z* is noise, *P* is probability distribution, and *E* is expectation.

The formula indicates that the training process of generative adversarial networks aims to minimize the generator loss and maximize the discriminator loss, that is, to continuously improve the data generation performance of the generator to achieve the goal of the discriminator being unable to recognize truth or falsehood.

### 2.2 Convolutional Neural Network (CNN)

**Fig 2:**
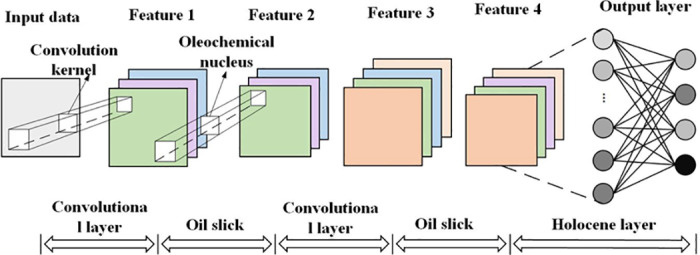
Convolutional Neural Network.

Convolutional Neural Networks (CNNs), as classic deep learning models, have been extensively applied to recognition tasks for data, images, and other signals [51]. Their basic structure, as illustrated in Fig 2, primarily functions through convolutional layers, pooling layers, and fully connected layers.

### 2.3 Long Short Term Memory Network (LSTM)

**Fig 3:**
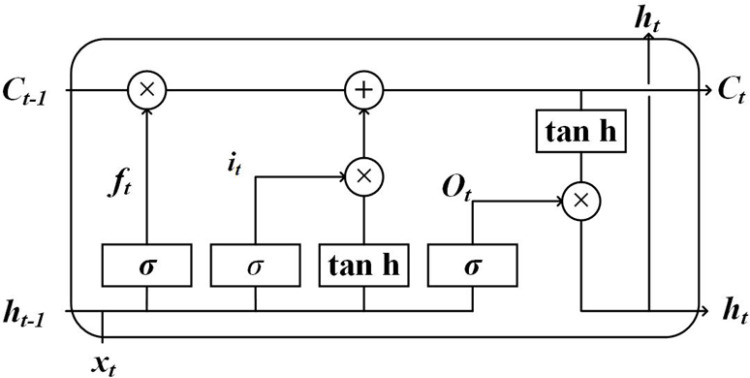
LSTM Network Structure.

LSTM is a variant of Recurrent Neural Networks (RNNs). Due to the vanishing gradient problem, RNNs possess only short-term memory. LSTM networks combine short-term and long-term memory through sophisticated gate controls, and to some extent, address the issue of decaying gradient information propagation, enabling the network to maintain long-term memory [52]. The structure of LSTM is illustrated in Fig 3.

Where *f*_*t*_ is the input gate, *i*_*t*_ is the forget gate, *O*_*t*_ is the output gate, *C*_*t*_ is the state of the neural unit at time *t*, *H*_*t*_ is the hidden layer state at time *t*; *σ* and *tanh* are activation functions.

## 3 Prediction framework for circular saw blade wear

**Fig 4:**
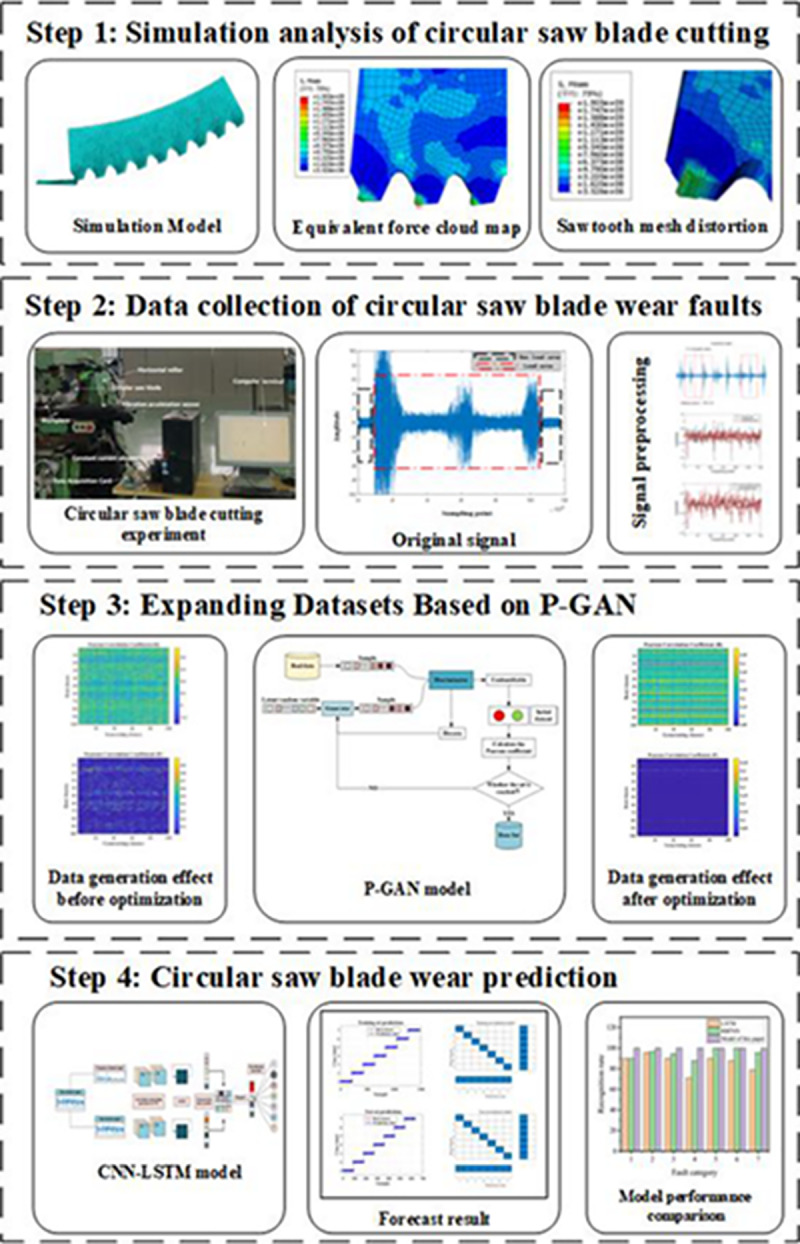
Prediction framework for circular saw blade wear.

The proposed circular saw blade wear prediction framework is shown in Fig 4, which includes the following four steps.

### 3.1 Simulation analysis of circular saw blade cutting stainless steel

**Fig 5:**
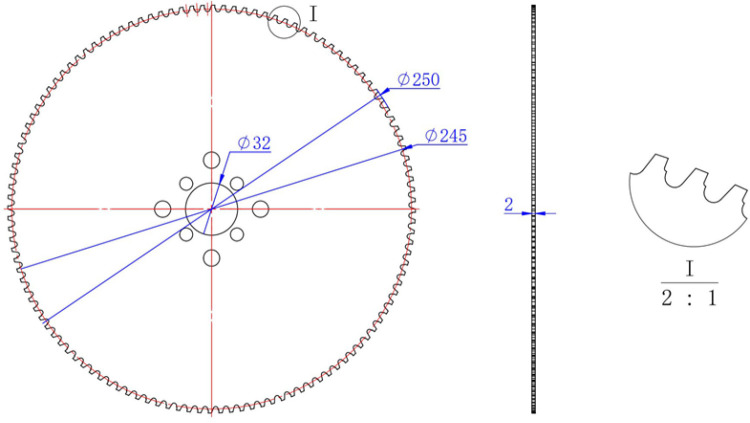
Basic structure model of saw blade.

Taking a specialized stainless steel cutting blade as the research object, the basic structural model of the blade is illustrated in Fig 5. The simulation focuses on the cutting of stainless steel by a circular saw blade. By observing part of the saw teeth, we can infer the locations on the blade that are susceptible to wear during actual operation. This analysis of tool wear fault types provides a reference for the classification of wear faults in circular saw blades.

The constitutive model parameters for the simulation of stainless steel cutting with a circular saw blade are presented in Table 1, the damage model parameters are shown in Table 2, and the basic physical properties of the cermet tool are listed in Table 3.

**Table 1:** J-C constitutive model parameters.

A(MPa)	B(MPa)	C	n	m
452	694	0.0067	0.311	0.996

**Table 2:** J-C damage model parameters.

d_1_	d_2_	d_3_	d_4_	d_5_
0.53	0.5	−6.8	0.014	0

**Table 3:** Basic physical properties of metal ceramic saw blades.

Density (gmm^-3^)	Yield strength (Pa)	Poisson’s ratio	Young’s modulus (Pa)	Thermal conductivity (gmms^-3^K^-1^)	Specific heat (mm^2^s^-2^K^-1^)
6.23 × 10^−3^	1.0215 × 10^9^	0.219	3.9948 × 10^11^	3.698 × 10^7^	5.0516 × 10^8^

**Fig 6:**
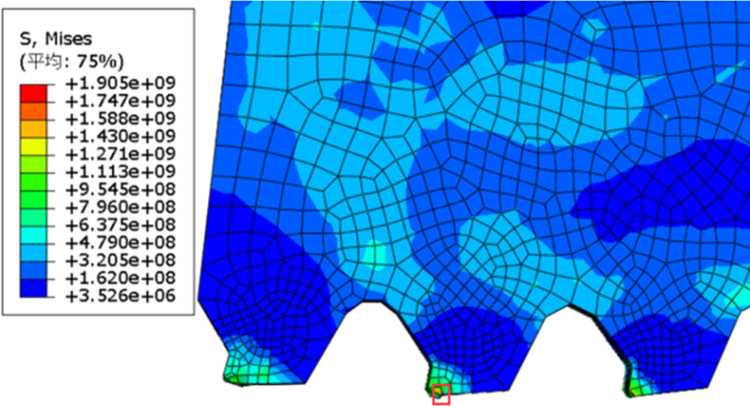
Equivalent force cloud map.

The stress experienced by the cutting tool during the cutting process is the primary cause of its wear and damage. Therefore, this simulation primarily observes the temperature generated by the cutting tool and the equivalent stress experienced by the tool during the stainless steel cutting process of the saw blade. Based on the parameters defined earlier, the simulation results are shown in Fig 6.

**Fig 7:**
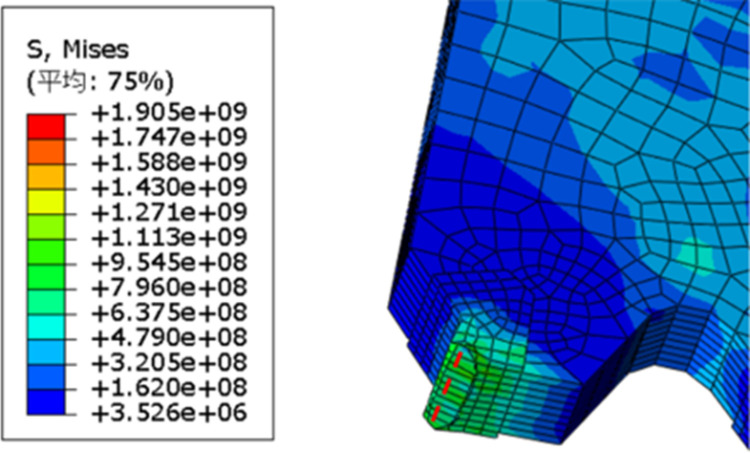
Sawtooth mesh distortion.

As shown in Fig 6, the maximum equivalent stress on the saw blade during the cutting process occurs at the main cutting edge and the flank face of the saw teeth, indicating that these areas are prone to wear during actual cutting. To further illustrate the damage to the saw blade, the changes in the saw tooth mesh in the simulation model were observed. Fig 7 depicts the distortion of the saw tooth mesh, with the red dashed line indicating the initial position of the main cutting edge of the saw tooth. After the cutting action occurs, the main cutting edge experiences high stress and wears, with the wear tending to spread towards the flank face. Therefore, considering the equivalent stress and node temperatures experienced by the saw blade during cutting, it is evident that the main cutting edge and the flank face are the primary wear areas of the saw blade. Additionally, when the saw teeth wear to a certain extent, the cutting force decreases, leading to increased stress, which may potentially cause the saw teeth to fracture.

Based on the above simulation results, the primary failure modes of the cermet circular saw blade are wear on the main cutting edge and fracture of the saw teeth.

### 3.2 Data collection and processing

**Fig 8:**
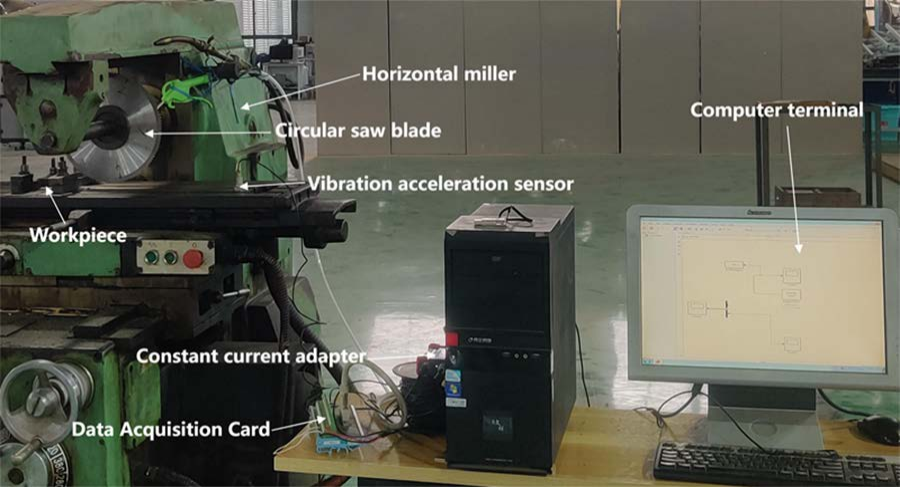
Experimental data collection platform.

An experimental platform was set up to cut 3 mm thick 304 stainless steel plates on an X6125 horizontal milling machine. The tool in use was a ceramic circular saw blade with an outer diameter of 250 mm, a thickness of 2 mm, and 120 teeth. During the cutting process, the spindle speed was set to 320r/min, the feed rate was 12 mm/min, and the cutting depth was 1 mm. Vibration signals were collected by using an IEPE vibration acceleration sensor and an Advantech PCI-1710 data acquisition card. The data has been recorded through MATLAB. The experimental platform is shown in Fig 8.

**Fig 9:**
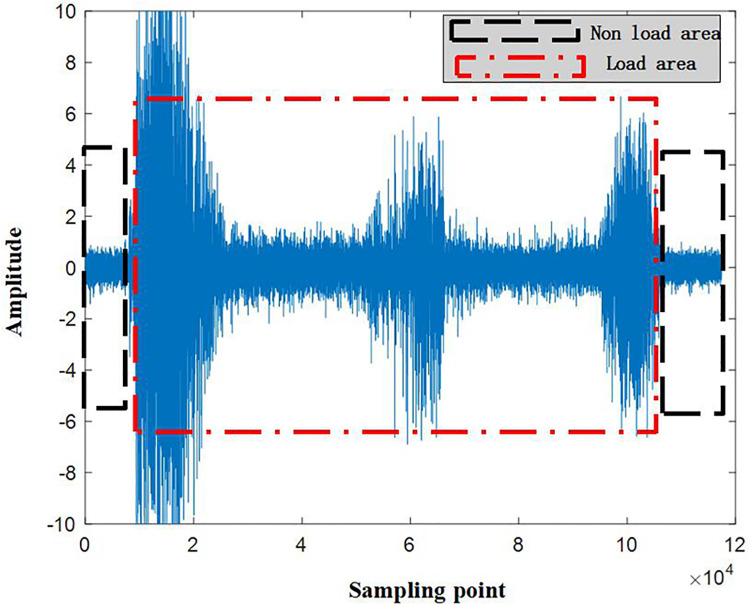
The raw samples of vibration signals.

**Fig 10:**
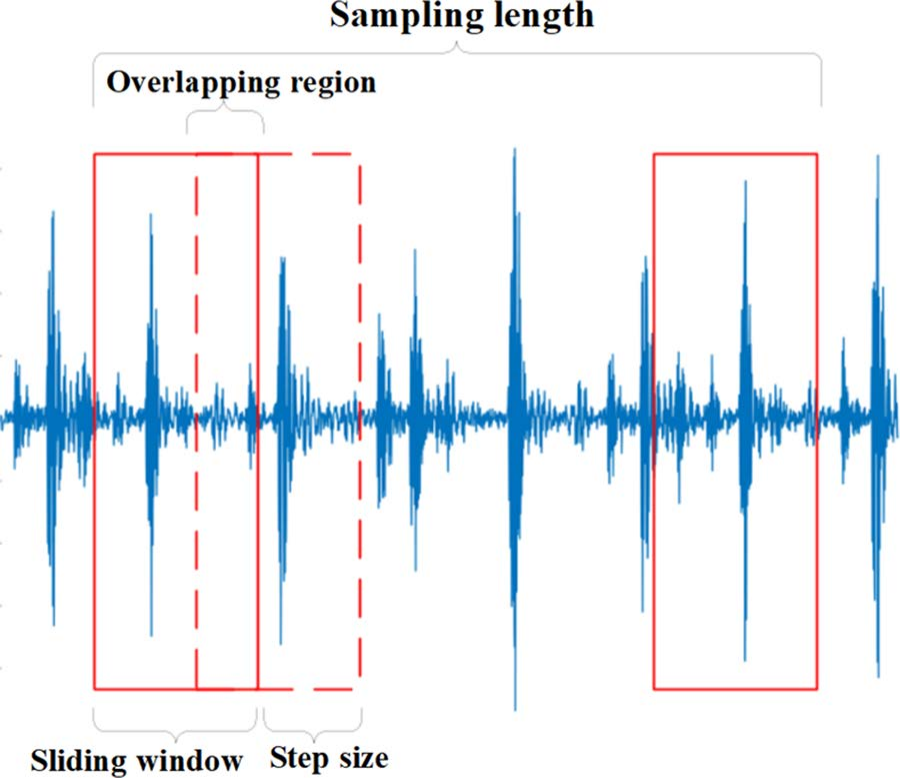
Overlapping sampling process.

The raw samples of the collected vibration signals are shown in Fig 9. It can be observed from the figure that the vibration signals collected during the experiment are long time series containing multiple repeated fault signals. Using such signals as raw data for training the recognition model can lead to data redundancy. Furthermore, there are some amplitude anomalies in the signal samples, which can easily result in inaccurate training of the circular saw blade wear prediction model. Therefore, the author removes the regions with abnormal amplitudes at the first step. Secondly, to enhance the correlation between data points, the original time series signals are divided through overlapping sampling, as illustrated in Fig 10.

**Fig 11:**
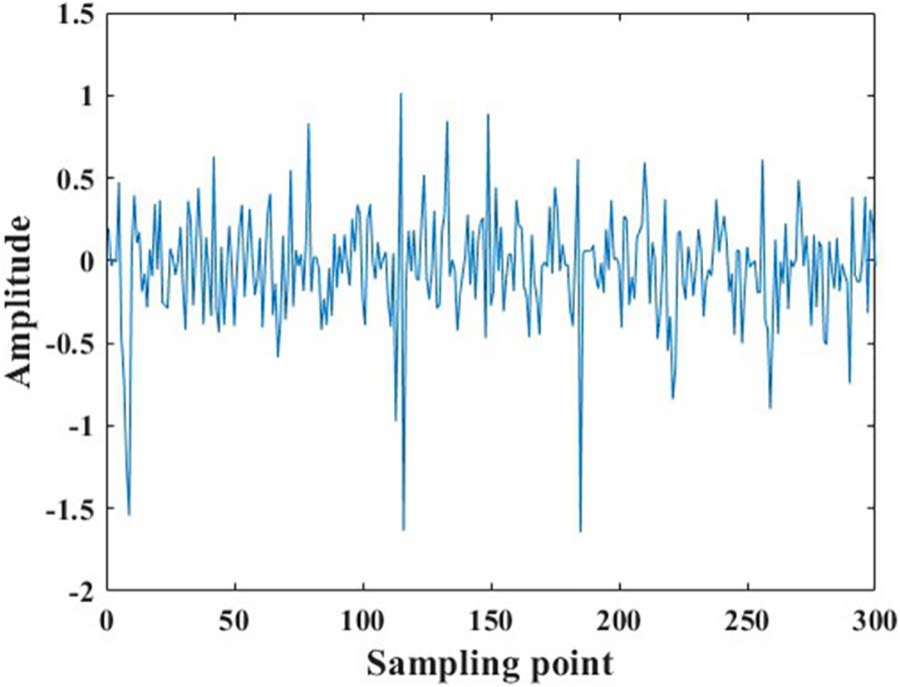
Overlapping sampling data samples.

**Fig 12:**
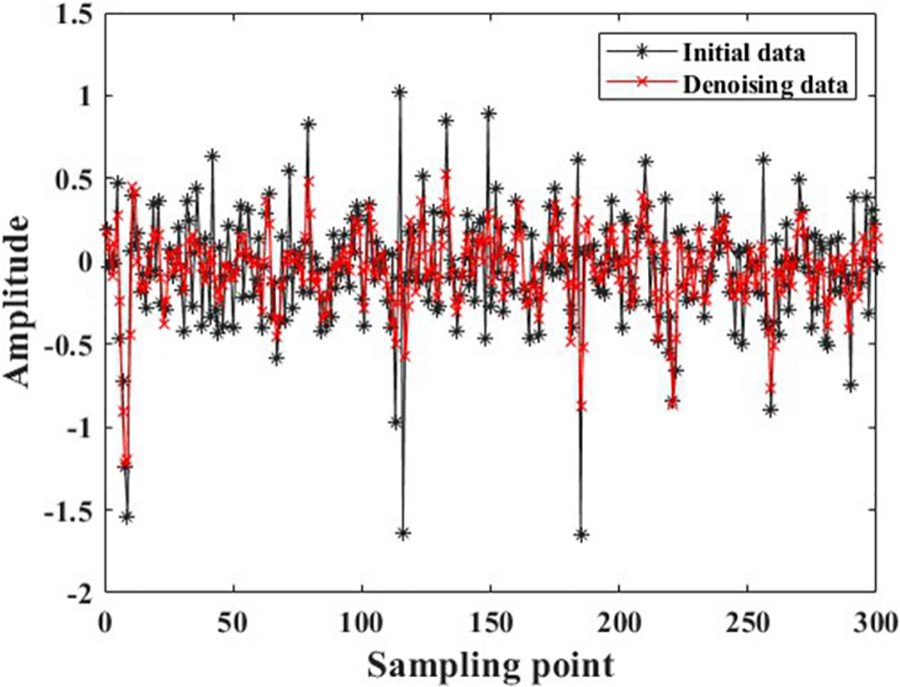
Comparison of data before and after denoising using single-layer wavelet transform.

**Fig 13:**
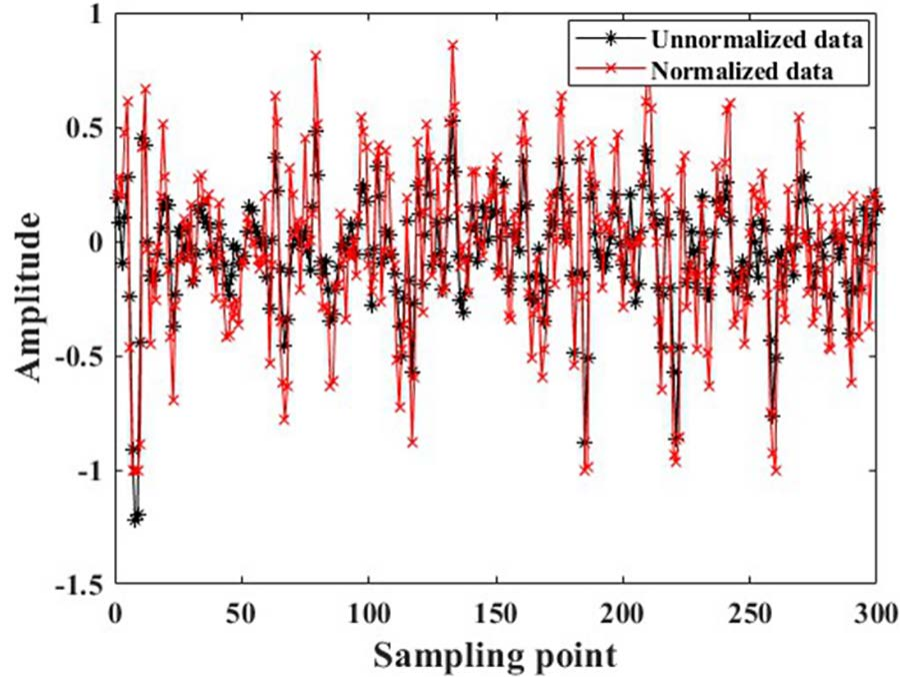
Comparison of data before and after normalization.

The data samples obtained through overlapping sampling still suffer from issues such as small data volume, noise contamination, and the presence of individual extremely large amplitudes, as shown in Fig 11. These issues may cause problems such as overfitting and convergence difficulties when recognition models are being trained which can result in low fault recognition rates for the models. Therefore, the author employed single-level wavelet transform to denoise the raw data, with a comparison of the data before and after denoising shown in Fig 12. Additionally, individual abnormal amplitudes can make it difficult for the recognition model to converge, causing faults in model training. The author further normalized the data with a comparison of the data before and after normalization shown in Fig 13. It can be observed that the normalized data maintains its amplitudes within the range of [−1, 1], eliminating the issue of large numerical discrepancies.

### 3.3 Optimizing the construction of generative adversarial network models

The Pearson correlation coefficient is an important statistical method for measuring the strength and direction of the linear relationship between two variables. It is simple and intuitive to calculate, easy to understand and interpret. Most importantly, compared to other methods, the Pearson correlation coefficient simultaneously calculates both the correlation and its significance, which is crucial for judging whether the correlation is significant.

**Fig 14:**
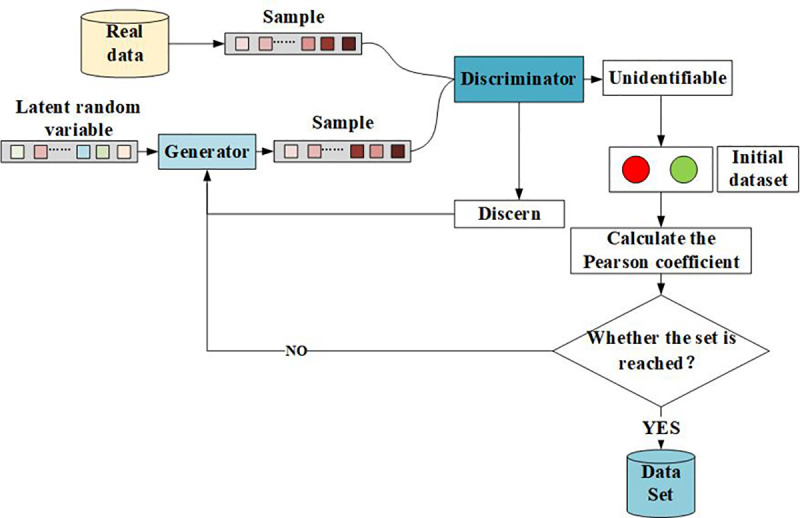
PCC-optimized GAN model.

In this paper, a Generative Adversarial Network (GAN) optimized using the Pearson Correlation Coefficient (PCC) is employed to evaluate the training performance of the network by analyzing the correlation between generated data and real data. The dataset collected is augmented using this PCC-optimized GAN. Identifying tool faults using deep learning algorithms often requires a vast amount of labeled data. However, in practical scenarios, obtaining a large number of labeled fault data is labor-intensive, resource-consuming, and challenging. Consequently, the issue of small sample sizes for fault data has been a major obstacle in developing fault recognition algorithms. To address this problem, extensive research has been conducted, and results indicate that augmenting experimental data through GANs is an effective solution. Nevertheless, the loss function of GANs suggests that, while theoretically the trained model can generate data with high similarity to real data, in practical applications, due to the lack of a performance evaluation criterion for the model, the authenticity of the generated data remains highly uncertain, i.e., the similarity between generated data and real data cannot be controlled. Therefore, this paper incorporates the PCC evaluation criterion into the GAN to improve the model’s data generation performance, as illustrated in Fig 14.

### 3.4 Construction of CNN-LSTM Model

**Fig 15:**
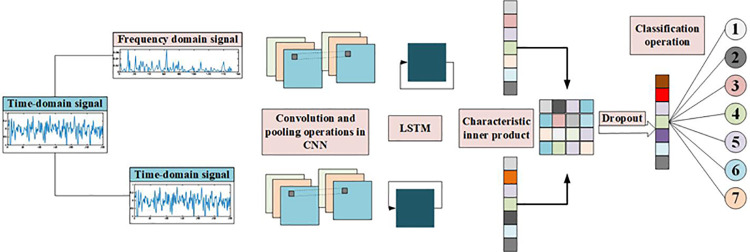
CNN-LSTM model based on dual feature fusion.

A circular saw blade wear prediction model based on dual feature fusion was constructed. In response to increasingly complex working conditions, the structure of tool wear prediction models has gradually shifted from single models to multi-model fusion, and most experimental results have also proven that multi-model combinations have higher tool wear prediction accuracy than single models. Circular saw blades are multi-edged tools with complex structures and work in complex environments, where vibration acceleration signals are susceptible to environmental noise interference. Therefore, to enhance the anti-interference ability of the tool wear prediction model and improve its prediction accuracy, this paper constructs a CNN-LSTM model based on Convolutional Neural Networks (CNNs) combined with LSTM, which can extract both spatial and temporal features of signals. The model structure is shown in Fig 15.

Firstly, the frequency domain data of the original time-domain signal is calculated using the fast Fourier transform, as shown in equation (2).


$$X(k)=\sum_{n=0}^{N-1}x(2n)W_{N}^{2kn}+W_{N}^{k}\sum_{n=0}^{N-1}x(2n+1)W_{N}^{2kn}$$ (2)

Where *X(k)* is the amplitude of the frequency domain signal, *k* is the frequency, *x(2n)* is the even sequence of the original signal, *x(2n + 1)* is the odd sequence of the original signal, *N* is the data volume of the original signal,$W_{N}=e^{-j\frac{2\pi }{N}}$, *k *= 1, 2, 3...... *N-*1.

The original time-domain signal is calculated using Equation 2 to obtain the corresponding frequency-domain signal, and combined with the original time-domain signal as the input signal for constructing the model in this paper. The two types of feature signals first extract preliminary features through convolution and pooling operations, and the convolution and pooling calculation processes are shown in equations (3) and (4).


$$X_{j}^{l}=f\left(\sum_{i\in M_{j}}X_{i}^{l-1}*\omega_{ij}^{l}+b_{j}^{l}\right)$$ (3)

where $X_{j}^{l}$ is the *j-th* feature of the *l-th* layer Output, *M*_*j*_ is the set of data in the input of the layer, $\omega_{ij}^{l}$ is the weight of the convolution kernel in the *l-th* layer, which is related to the input $x_{i}^{l-1}$ corresponding to the weight matrix, $b_{j}^{l}$ is the bias of the *j-th* convolution kernel in the *l-th* layer, *f()* is the nonlinear function of the network, * is the convolution operator.

Commonly used pooling operations are maximum pooling and average pooling, this paper selects the maximum pooling operation, the calculation process is as follows.


$$a_{ij}^{k}=\underset{u\in D_{j}^{k-1}}{\max}[x_{i}^{k-1}(u)]$$ (4)

where $a_{ij}^{k}$ is the output value of the maximum pooling layer, $D_{j}^{k-1}$ is the pooling region, $x_{i}^{k-1}$ is the *i-th* feature map output from the *k-1st* convolutional layer.

After the time domain and frequency domain signals are convolved and pooled, the feature data extracted from them are processed by the long and short-term memory neural network, and the weights obtained from the two processes are inner-processed, and the weights obtained from the two kinds of data are combined to classify the data, and then get the type of wear state to which the data belongs.

## 4 Experiments and model validation

### 4.1 Experimental design and data preparation

In this paper, the criteria for discarding circular saw blades are defined as when the wear on the top edge of the saw tooth reaches 1/4–1/3 of the tooth width or when more than two adjacent saw teeth are fractured. To define the wear state of the saw blades used in this paper and to refine the degree of tooth wear, the tooth width of the saw blades employed was measured to be 1.32 mm. Based on the discard criteria for wear, wear values exceeding 0.4 mm are considered as the standard for discarding worn saw blades in this paper, and the degree of wear of the circular saw blades is refined according to intervals of 0.1 mm in wear values. The types of circular saw blade failures defined in this paper are shown in Table 4.

**Table 4:** The types of circular saw blade failures.

Label	Types of circular saw blade failures
1	Normal
2	Wear 0–0.1 mm
3	Wear 0.1–0.2 mm
4	Wear 0.2–0.3 mm
5	Wear 0.3–0.4 mm
6	Wear over 0.4 mm
7	serrated breakage

**Fig 16:**
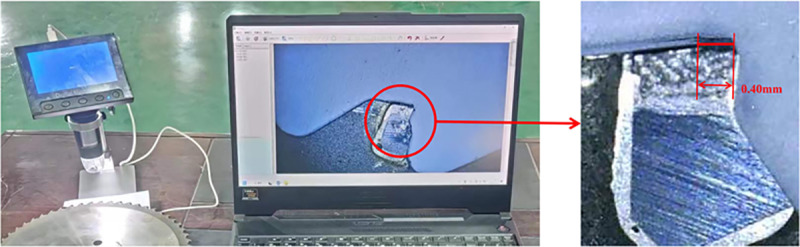
Measurement of saw blade wear value.

Considering the subtle variations in tooth size during the manufacturing process, five saw teeth were randomly selected as the target teeth for measuring wear, and the average wear value of these five teeth was taken as the wear state of the saw blade. The wear values of the circular saw blades were measured using an optical electronic magnifier, as shown in Fig 16. The measurement results of the wear values for each tooth state are presented in Table 5.

**Table 5:** Calibrated serration wear values for each condition.

Sawtooth	state 2	state 3	state 4	state 5	state 6
**1**	0.09mm	0.21mm	0.24mm	0.35mm	0.43mm
**2**	0.07mm	0.19mm	0.26mm	0.32mm	0.44mm
**3**	0.04mm	0.15mm	0.24mm	0.30mm	0.40mm
**4**	0.04mm	0.16mm	0.25mm	0.33mm	0.37mm
**5**	0.06mm	0.17mm	0.27mm	0.31mm	0.41mm

Fig 16 illustrates the measured wear value of calibrated tooth 3 in state 6. It is evident from the figure that during the cutting of 304 stainless steel, the wear on the cermet saw blade used in this paper extends from the main cutting edge towards the flank face, indicating that the simulation model constructed in Section 3.1 aligns with reality and that the saw blade states defined therein are feasible. The wear measurement values presented in Table 5 reveal that the wear degree of calibrated tooth 1 at the onset of cutting is significantly higher than that of the other teeth, suggesting that this tooth was slightly larger in size during manufacturing. However, by calculating the average wear value of the five calibrated teeth, it can be observed that their wear states all fall within the wear degree range set by this paper. The average wear values for wear states 2–6 are 0.06 mm, 0.176 mm, 0.252 mm, 0.322 mm, and 0.41 mm, respectively.

### 4.2 Model training and performance evaluation

#### (1) Optimizing the dataset expansion of generative adversarial networks.

**Fig 17:**
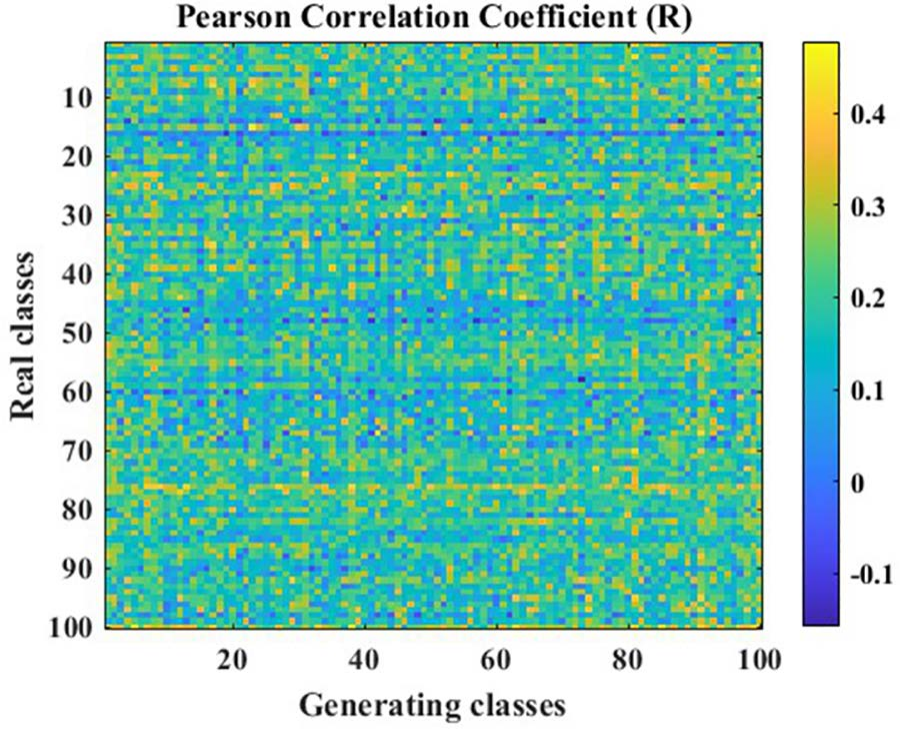
PCC R-value cloud map.

**Fig 18:**
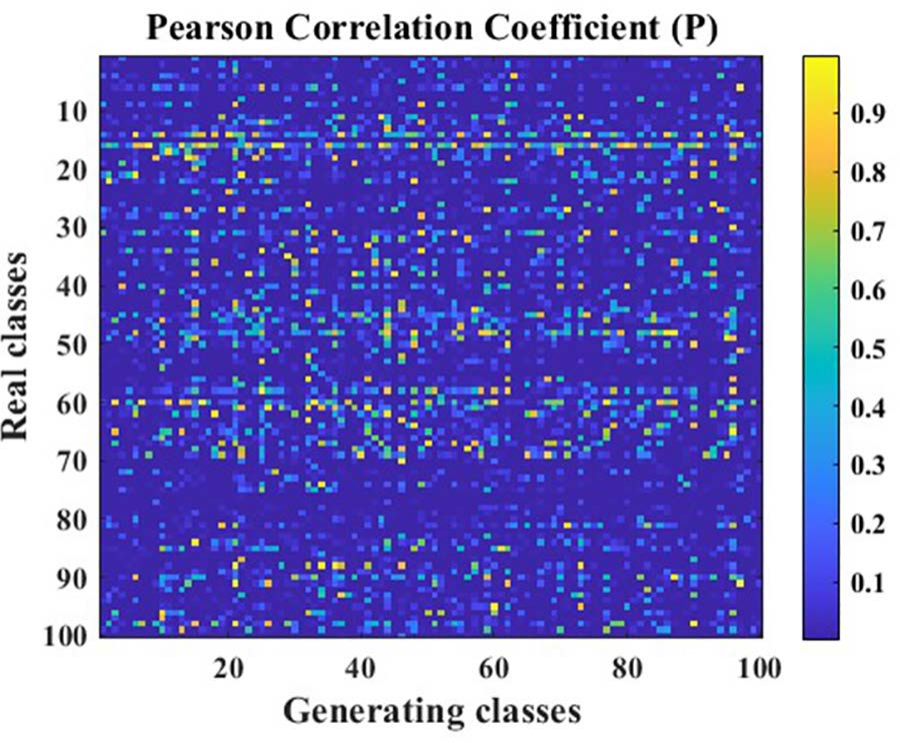
PCC P-value cloud map.

In this paper, vibration acceleration signals from seven fault states of circular saw blades were collected. A Generative Adversarial Network (GAN) optimized using the Pearson Correlation Coefficient (PCC) was employed to augment each state’s dataset, and the data generation effectiveness before and after optimization was compared. The data generation performance of the traditional GAN on the data utilized in this paper is illustrated in Fig 17, 18.

In Fig 17, 18, the Pearson Correlation Coefficient (PCC) between data generated by a traditional Generative Adversarial Network (GAN) and real data is presented. The X-axis represents the labels of 100 generated data points, while the Y-axis represents the labels of 100 real data points. The PCC is a crucial metric for evaluating whether there is a correlation between datasets. The R-value indicates the correlation between the data, while the P-value is used to test the null hypothesis of no correlation against the alternative hypothesis of non-zero correlation. When the P-value is less than the significance level, it indicates that the null hypothesis of no correlation between the data is rejected, i.e., the correlation identified by the R-value exists. Typically, the significance level is set at 0.05. The correlation hypotheses based on different R-value ranges are shown in Table 6.

**Table 6:** Correlation assumptions identified for different R-value ranges.

R-value range	Data relevance assumptions
0.0 ≤ R ≤ 0.2	Very weakly correlated or uncorrelated
0.2 ≤ R ≤ 0.4	Relevance
0.4 ≤ R ≤ 0.6	Moderately relevant
0.6 ≤ R ≤ 0.8	strong correlation
0.8 ≤ R ≤ 1.0	very strong correlation

Based on the correlation hypotheses identified by the different R-value ranges in Table 6, the partial data generated by the traditional Generative Adversarial Network (GAN) in Fig 17 exhibits a weak or non-existent correlation with the real data. To further demonstrate the overall correlation between the data generated by the traditional GAN and the real data, the average R-value was calculated to be 0.1794, indicating a poor overall correlation between the generated data and the real data. Additionally, the P-values in Fig 18 reveal that the identified correlation does not exist in some cases, suggesting that the correlation in the data generated by the traditional GAN is unstable. In other words, it is uncertain whether there is a correlation between the generated data and the original data.

By incorporating Pearson correlation calculations into the Generative Adversarial Network (GAN), we can further assess the quality of the generated data and thereby enhance the data generation capability of the generator. The hyperparameter settings for the PCC-optimized GAN are presented in Table 7.

**Table 7:** The hyperparameter settings for the PCC-optimized GAN.

MaxEpochs	Generator Learning Rate	Discriminator learning rate	gradient decay factor	squared gradient decay factor	Relevance score
100	0.0002	0.0002	0.5	0.999	0.3

**Fig 19:**
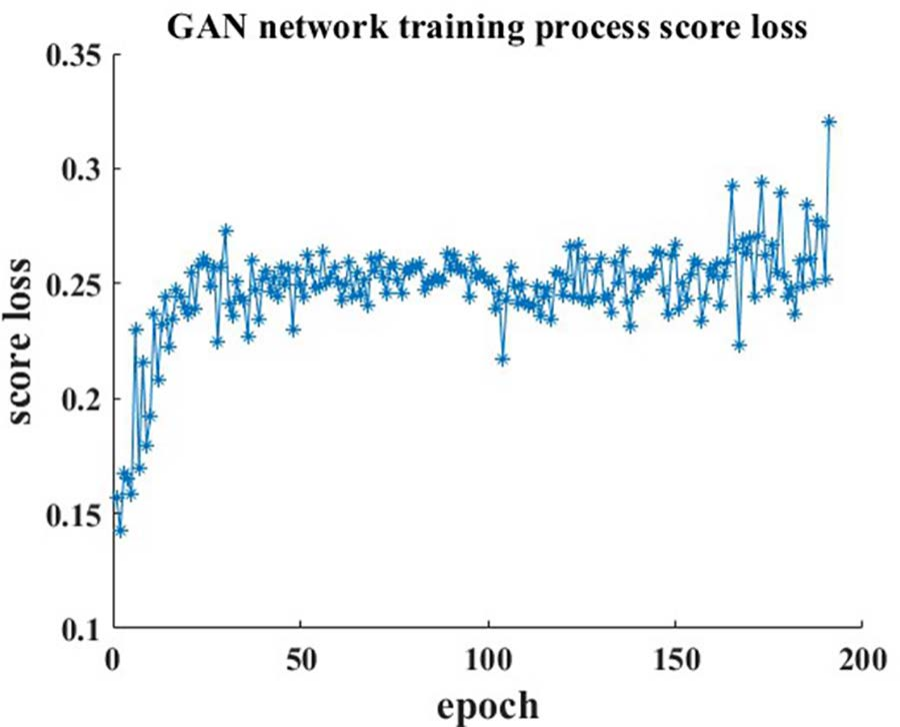
Analysis of the effect of correlation coefficient.

In Table 7, the learning rates of the generator and discriminator effectively control their learning progress, better assisting the network in converging to a global optimal solution. The setting of the decay factor can effectively regulate the magnitude of the learning rate, stabilizing the model and enabling it to reach an extreme value efficiently during the later stages of network iteration. The incorporation of correlation settings ensures stable data generation quality by the network and maintains an appropriate correlation between the generated data and real data. The hyperparameters are determined by calculating the correlation coefficient between two sets of real data. Their roles during the training process are illustrated in Fig 19.

As shown in Fig 19, during the training process, the model continuously calculates the correlation between the generated data and real data. When the correlation reaches a desired level, the network training is halted. This approach effectively stabilizes the data generation quality of the model, preserving sufficient correlation while avoiding overfitting.

**Fig 20:**
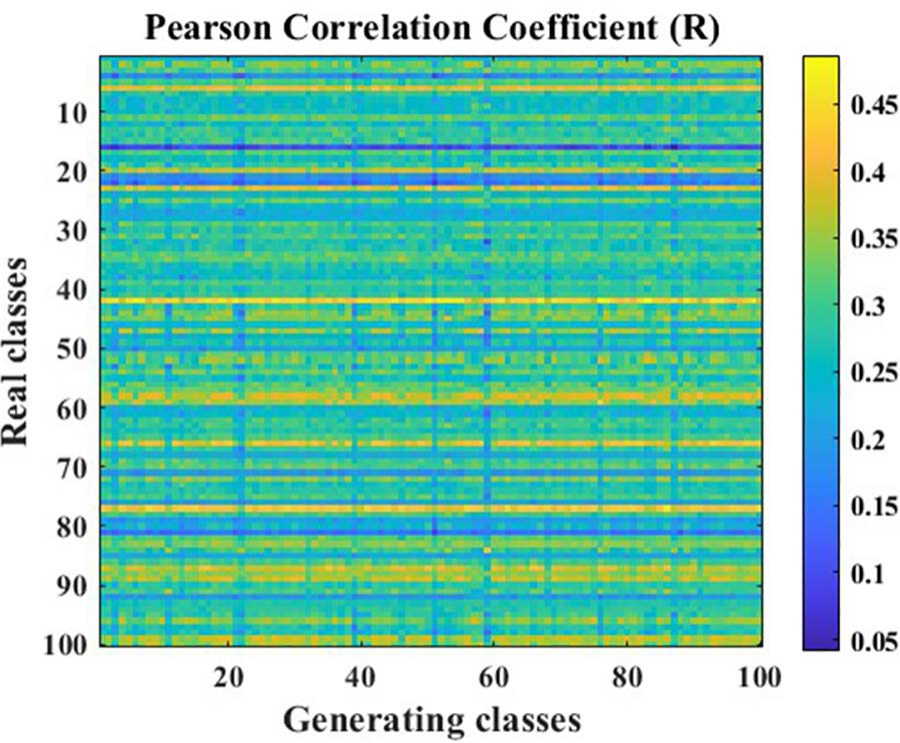
PCC R-value cloud map.

**Fig 21:**
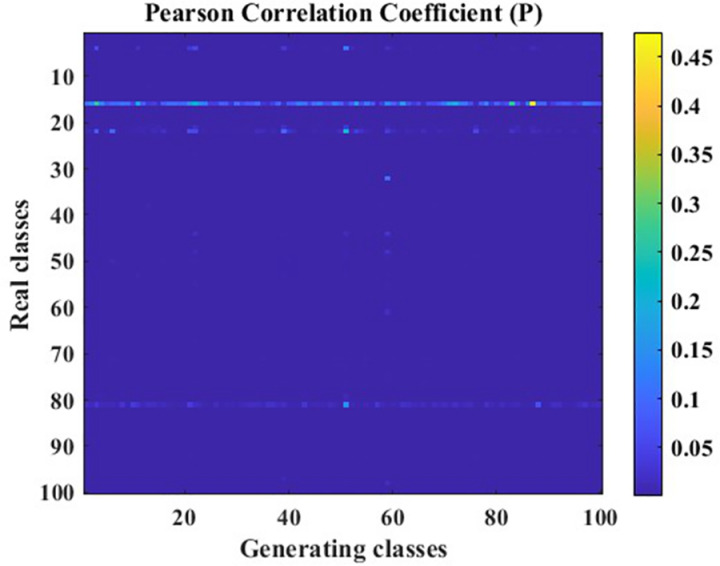
PCC P-value cloud map.

The data generation effectiveness of the Generative Adversarial Network (GAN) model optimized using the Pearson Correlation Coefficient is illustrated in Fig 20, 21.

As shown in Fig 20, 21, after optimization, the correlation between the generated data and real data has been significantly improved, with an average correlation coefficient of approximately 0.3. This indicates that there is a correlation between the generated data and real data, which is an improvement of 43% compared to before optimization. Furthermore, the calculated P-values reveal that, except for a few outliers where the P-value is above the significance level, most P-values are below the significance level. This suggests that the correlation identified by the R-value exists and is relatively stable.

The aforementioned results demonstrate that, after optimization using the method proposed in this paper, the data generation capability of the Generative Adversarial Network (GAN) has been significantly enhanced, and there is a correlation between the generated data and real data. Therefore, based on the optimized GAN model constructed in this paper, the experimental data has been expanded, with the data volume under each condition increased to 300, resulting in a total data volume of 2,100.

#### (2) Performance evaluation of CNN-LSTM model prediction.

To verify the superiority of the dual feature fusion CNN-LSTM model for identifying tool wear states during the cutting of stainless steel by circular saw blades, the model’s performance was tested using the complete data set expanded by the optimized GAN, and a confusion matrix was plotted. The entire data set was divided into a training set and a test set in a 7:3 ratio, with 1470 data points in the training set and 630 data points in the test set, with the same number of data points for each circular saw blade wear state. Before training the model, the model’s hyperparameters were adjusted and set. The relevant hyperparameter settings for the recognition model are shown in Table 8.

**Table 8:** The relevant hyperparameter settings for the recognition model.

MaxEpochs	DropoutLayer	LearnRateDropFactor	L2-Regularization	InitialLearnRate
120	0.35	0.004	0.08	0.001

The setting of the dropout factor contributes to making the features learned by the network more robust. The configuration of the learning rate and learning rate decay factor facilitates the model in converging to an optimal solution during the training process. Meanwhile, the adjustment of the L2 regularization parameter helps prevent overfitting and enhances the training effectiveness of the model.

**Fig 22:**
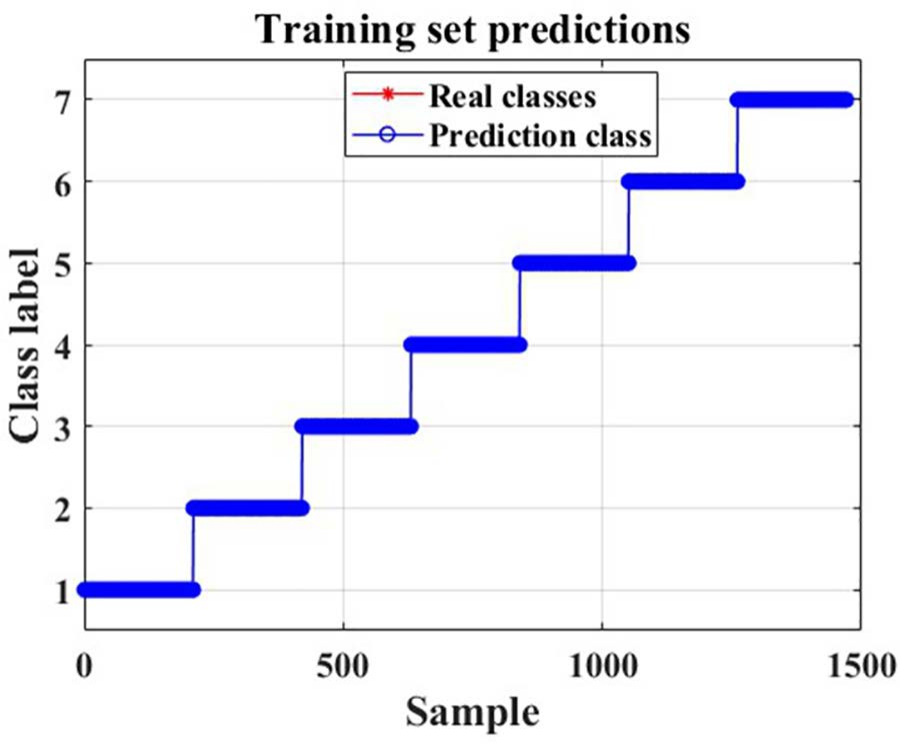
Training set prediction results.

**Fig 23:**
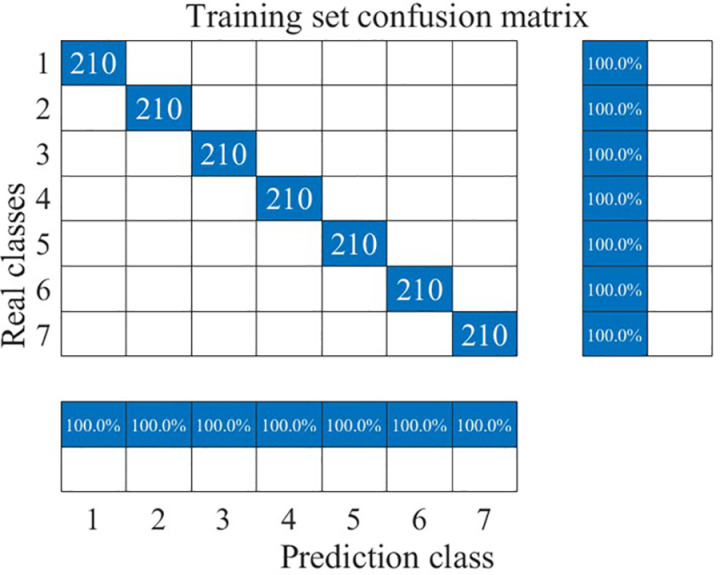
Training set identification confusion matrix.

**Fig 24:**
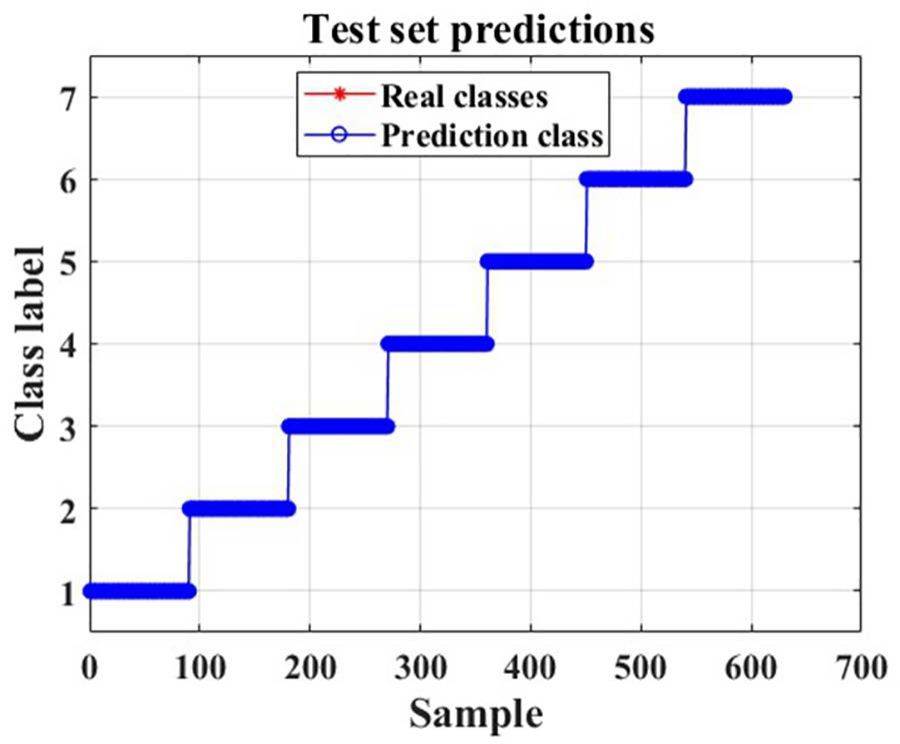
Test set prediction results.

**Fig 25:**
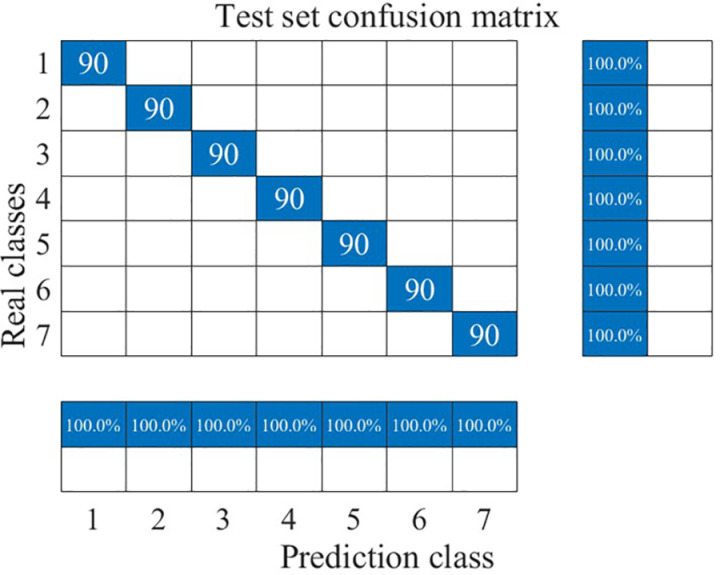
Test set identification confusion matrix.

The recognition results for the training and test sets are shown in Figs 22–25.

As can be seen from Fig 22 and Fig 25, the CNN-LSTM model using dual-feature fusion demonstrates a high accuracy in predicting the wear state of circular saw blades. The recognition rates for each wear state of the circular saw blades in both the training and testing sets reach 100%, and there is no occurrence of overfitting or underfitting during the training process. This result indicates that the circular saw blade wear state prediction model constructed in this paper exhibits excellent performance and can effectively address the issue of tool wear prediction for circular saw blades cutting stainless steel.

### 4.3 Comparative analysis of model performance

**Fig 26:**
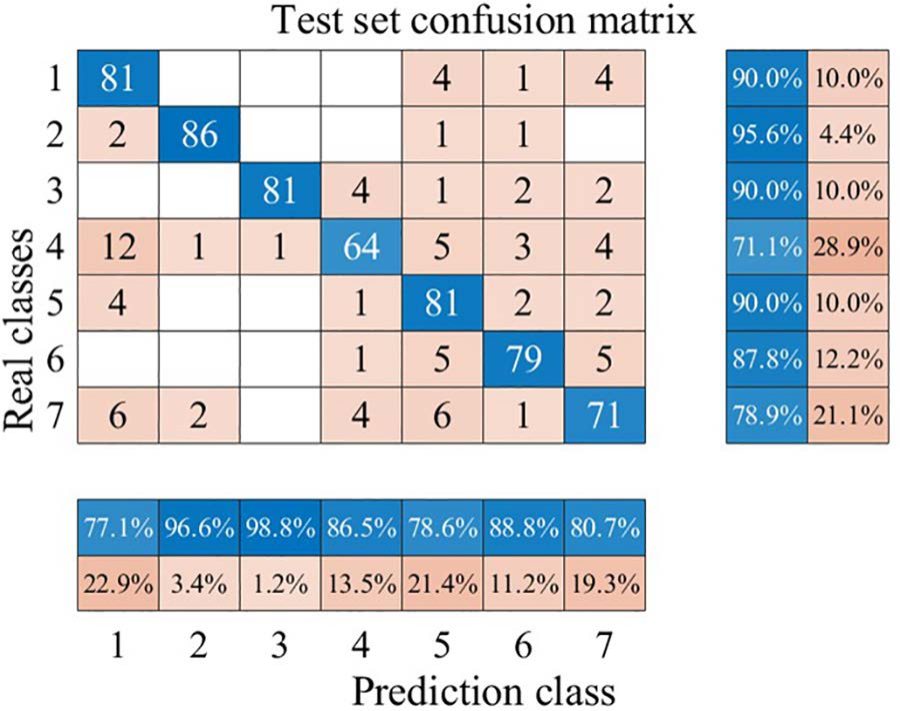
Confusion Matrix of LSTM.

**Fig 27:**
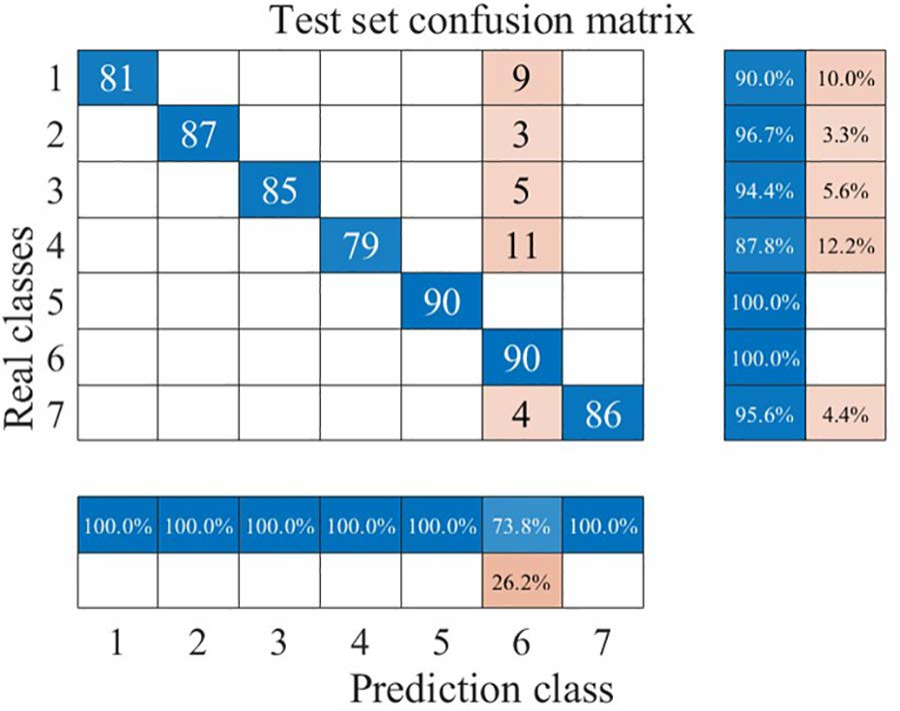
Confusion Matrix of RBFNN.

To further demonstrate the superiority of the circular saw blade wear prediction model constructed in this paper, we trained and tested the recognition performance of Long Short-Term Memory (LSTM) neural networks and Radial Basis Function Neural Networks (RBFNNs) for circular saw blade wear states using the same total dataset of time-domain signals. The ratio of the training set to the testing set was also maintained at 7:3 for both models. The wear state recognition results for both models are shown in Fig 26, 27.

**Fig 28:**
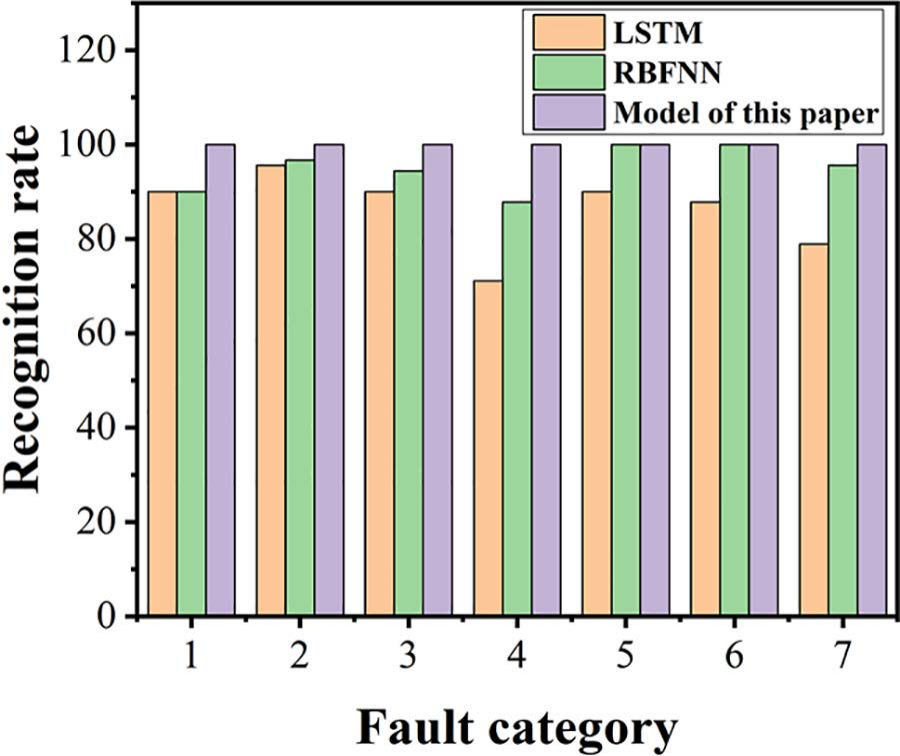
Performance Comparison of Models.

As can be seen from Fig 26, 27, both the Long Short-Term Memory (LSTM) neural network and the Radial Basis Function Neural Network (RBFNN) exhibit lower accuracy in recognizing the wear states of circular saw blades in the dataset used in this paper compared to the model constructed in this study. Specifically, the average wear state recognition accuracy of the LSTM network is 86.2%, while that of the RBFNN is 94.9%. To visually represent the wear state recognition performance of the models under different conditions, the author has compiled the wear state recognition rates for all three models, as shown in Fig 28.

The comparison results indicate that, when using the same dataset, the model constructed in this paper demonstrates superior recognition performance across various wear states of circular saw blades compared to other models. The recognition rate of this model is improved by an average of 5%−15% relative to traditional single deep learning models, exhibiting excellent wear prediction capabilities and meeting the requirements for intelligent prediction of circular saw blade wear faults.

## 5 Conclusion

A circular saw blade wear prediction method combining generative adversarial networks and CNN-LSTM models is proposed to achieve intelligent prediction of circular saw blade wear status under complex working conditions. This method uses a GAN optimized by Pearson correlation calculations to expand the quantity of fault data sets and simultaneously uses the frequency domain signals and time domain signals of circular saw blade vibration data as model inputs to achieve accurate prediction of circular saw blade wear status. The advantages of the model is verified through experiments. The main conclusions are as follows:

(1) Through ABAQUS simulation analysis, the main fault types during the cutting process of stainless steel shells of spent fuel assemblies by circular saw blades were identified as wear on the main cutting edge and tooth fracture, and the wear states of circular saw blades were classified into seven categories.
(2) By incorporating Pearson correlation calculations into the GAN, the correlation between generated data and actual data has been obviously improved and stable so that successfully expands the data volume of each fault state to 300 samples, with a total data volume of 2100 samples, and effectively solves the problem of small and unstable data for circular saw blade wear faults under complex working conditions.
(3) The dual feature fusion CNN-LSTM model can simultaneously extract frequency domain signals and time domain signals of vibration data, achieving efficient and accurate circular saw blade wear state recognition with an accuracy rate of 100%, which is much higher than that of LSTM (86.2%) and RBFNN (94.9%).

Despite the high accuracy achieved in circular saw blade wear prediction in this study, the experimental data mainly originated from specific types of circular saw blades and stainless steel materials, so the generalization ability of the model needs further verification. Meanwhile, in practical applications, sensor installation and data collection may be limited by the environment, and how to optimize data collection methods to improve model performance still requires further research. We will continue to expand the types and scale of experimental data, including using different types of circular saw blades and materials, to verify the generalization ability of the model. Exploration of data expansion methods such as simulation and digital twins.
